# 987. Identification of Fungal Pathogens In Formalin-Fixed Paraffin-Embedded (FFPE) Tissues by Targeted Next-Generation Sequencing (NGS) Technology

**DOI:** 10.1093/ofid/ofad500.042

**Published:** 2023-11-27

**Authors:** Marissa C Totten, Christopher P Morris, Pramila Ariyaratne, Tony Zhang, Sean Zhang, Charlie Lee

**Affiliations:** Johns Hopkins School of Medicine, Baltimore, MD; National Institute of Allergy and Infectious Disease, National Institutes of Health, Bethesda, Maryland; Vela Diagnostics, Fairfield, New Jersey; Vela Diagnostics, Fairfield, New Jersey; Johns Hopkins Hospital, Baltimore, Maryland; Vela Diagnostics, Fairfield, New Jersey

## Abstract

**Background:**

Histological presence of fungal organisms associated with tissue damage is the hallmark for invasive fungal diseases. Rapid and accurate identification (ID) of these fungal pathogens present is critical for initiating appropriate treatment without delay. We investigated an automated sample-to-answer targeted NGS workflow for the ID of fungi directly from FFPE tissues.

**Methods:**

170 FFPE tissues (93 cases) from 2002 to 2022 at Johns Hopkins Hospital were selected based on histologic findings of the presence of fungal pathogens. Samples were run by PathoKey SQ Flex assay, (Vela Diagnostics) which includes automated extraction, library preparation and emulsion PCR followed by NGS on a Sentosa SQ301 and analyzed using Vela’s curated database. Two negative controls (NCs) were included on each run. To compare NGS results, 51 samples were extracted using the MagMAX™ FFPE DNA/RNA Ultra Kit (Applied Biosystems) followed by pan-fungal PCR (ITS1/ITS2/D1D2) and Sanger sequencing (SS) and analyzed through NCBI nucleotide blast for ID.

**Results:**

116/170 (68%) samples had culture results: 69/116 (59%) were concordant with Vela NGS (VS) ID to the genus/species level; 18/28 (64%) samples were concordant with SS ID to the genus/species level. Among 54 samples with no/negative culture results: 26/54 (48%) were presumptive Mucorales, of which 23/26 (88.5%) were supported by VS ID and 5/12 (42%) supported by SS ID; 12/54 (22%) were presumptive aspergillus-like hyphae fungi, of which 10/12 (83%) were supported by VS ID and 4/9 (44%) supported by SS ID. 15/170 (8.8%) samples had no VS ID due to QC failures, and 15/26 (58%) with no VS ID had no organism seen or scant amount of fungal load in the histology slide.

**Conclusion:**

VS detected fungal organisms in 76% (129/170) of FFPE tissues, of which it correctly ID-ed pathogens down to the genus/species level in 94% (102/129) of samples. VS is more sensitive to pathogen ID than SS. Samples with discordant or no results by VS were mostly due to small tissue input or low fungal loads present in histology. Environmental or human skin flora contamination reads overshadowing possible pathogens were observed in few cases; therefore, it is critical to include NCs in each run to distinguish them from clinically significant fungal pathogens.

**Disclosures:**

**Marissa C. Totten, MA**, Vela Diagnostics: Grant/Research Support **Pramila Ariyaratne, n/a**, Vela Diagnostics: Employee **Tony Zhang, n/a**, Vela Diagnostics: Employee **Sean Zhang, MD, PhD**, Applied BioCode: Grant/Research Support|IMMY Diagnostics: Grant/Research Support|KARIUS: Advisor/Consultant|Pearl Diagnostics: Grant/Research Support|Scanogen: Grant/Research Support|T2 biosystems: Advisor/Consultant|Vela Diagnostics: Grant/Research Support **Charlie Lee, Ph.D**, Vela Diagnostics: Employee

